# Sero-diagnostic evaluation of *Toxoplasma gondii* recombinant Rhoptry antigen 8 expressed in *E. coli*

**DOI:** 10.1186/1756-3305-7-297

**Published:** 2014-07-01

**Authors:** Parthasarathy Sonaimuthu, Mun Yik Fong, Ramaswamy Kalyanasundaram, Rohela Mahmud, Yee Ling Lau

**Affiliations:** 1 Department of Parasitology, Faculty of Medicine, University of Malaya, 50603 Kuala Lumpur, Malaysia; 2 Department of Biomedical Sciences, College of Medicine, University of Illinois, Rockford1601 Parkview Avenue, Rockford, Illinois 61107, USA

**Keywords:** *Toxoplasma gondii*, ROP8, Western blot, Expression

## Abstract

**Background:**

*Toxoplasma gondii* infects all warm-blooded animals, including humans. Early diagnosis and determining the infective stage are critical for effectively treating immunosuppressed individuals and pregnant women with toxoplasmosis. Among the rhoptry proteins of the parasite, Rhoptry protein 8 (ROP8), is known to be expressed during the early stages of *T. gondii* infection and is involved in parasitophorous vacuole formation. In this study, we have investigated the diagnostic efficacy of recombinant ROP8 (rROP8).

**Methods:**

The ROP8 gene was cloned into pCOLD I DNA vector and expressed as a soluble recombinant antigen in Escherichia coli. Expressed ROP8 protein was evaluated using western blot method.

**Results:**

Western blot analysis of purified rROP8 antigen using 200 T. gondii-infected human serum samples, as well as non-infected serum controls, allowed for the successful identification of toxoplasmosis-positives, yielding a 90% sensitivity and 94% specificity.

**Conclusion:**

Our findings indicated that rROP8 antigen expressed in *E. coli* was able to detect toxoplasmosis in infected human serum with specificity and sensitivity suggesting that rROP8 antigen represents a valid diagnostic marker for toxoplasmosis.

## Background

*Toxoplasma gondii* is an obligate intracellular parasite that infects about one third of the human population [1-3]. The incidence of *T. gondii* infection varies based on geographical location due to environmental conditions, cultural habits, and hygiene standards [2,4,5]. Although acute toxoplasmosis is typically asymptomatic in HIV/AIDS patients, chronic infection in immunocompromised individuals can lead to life threatening encephalitis [6,7]. In addition, toxoplasmosis is known to cause severe complications in pregnant women, including miscarriage.

Commercially available serological kits for the diagnosis of toxoplasmosis are usually based on total antigens isolated from *in vivo* or *in vitro* cultured *T. gondii.* Notably, production of these antigens is expensive, and they commonly contain host cell-derived contaminants [8-10]. In addition, the use of live parasites during antigen preparation can lead to health risks. In order to overcome these limitations, recombinant DNA technology has been employed for the production of antigens [11,12]. In fact, the use of this cost effective method allows for the purification of stage-specific *T. gondii* antigens, which can be used to differentiate acute and chronic infections [13]. In this regard, many antigenic genes from *T. gondii* have recently been cloned and expressed using various systems. Also, several reports have described the successful use of recombinant antigenic proteins to detect antibodies against *T. gondii*[8,11,14-19]. These previous studies analyzed recombinant antigens both individually and in combination to increase diagnostic sensitivity.

Among the rhoptry antigens, rhoptry protein 2 (ROP2) family proteins have been extensively studied. Rhoptry protein 8 (ROP8), which is a member of the ROP2 family, is expressed in both tachyzoites and bradyzoites of *T. gondii*[20]. Rhoptry antigens are involved in host cell invasion as well as parasitophorous vacuole membrane (PVM) formation [21]. Therefore, several rhoptry antigens (ROP1, 2, 8, 16, and 18) have been tested as potential vaccine candidates. While ROP1 and ROP2 were evaluated with regard to serological detection of *T. gondii*-infected serum samples, ROP8 has not been assessed as a diagnostic candidate. Therefore, in the present study, we have expressed recombinant ROP8 (rROP8) and investigated its efficacy as a diagnostic marker for toxoplasmosis in humans.

## Methods

### Parasite

The tachyzoites of *T. gondii* were propagated using HFF cells that were cultured and maintained in complete DMEM medium (2 mM glutamine supplemented with 10% (v/v) FBS, 5 μg/ml streptomycin and 5 U/ml penicillin) at 37°C with 5% CO_2_. The parasites were egressed out of the host cells by passing through a 25 G needle and purified using a 3 μm pore size polycarbonate membrane filter. The tachyzoites were harvested by centrifuging at 300 g for 15 min and suspended in appropriate buffer prior to use.

### Recombinant plasmid constructions

The genomic DNA of tachyzoites (RH strain) was isolated using TriZol reagents (Invitrogen, USA). The *ROP8* gene (Genbank ID: AF011377) was amplified from the genomic DNA using gene specific primers (forward primer 5^’^CCC*AAGCTT*AGCATGGAATTTTCTGTGTTACG 3’ and reverse primer 5^’^ CG*GAATTC*TCATGCCGGTTCTCCATCAG 3’containing the *Hind*III and *EcoR*1 restriction sites as italicized). The PCR product was purified and cloned into the pCOLD I DNA vector plasmid at the specified restriction site. The resulting recombinant construct was transformed into BL21 (DE3) *E. coli* expression host cells. Recombinant clones were screened and verified by DNA sequencing.

### Expression of recombinant protein

A single colony of fresh transformant of the recombinant construct was inoculated in 5 ml of Luria Bertani (LB) broth with Ampicillin (100 mg/ml) and Chloramphenicol (34 mg/ml) and grown overnight at 37°C, with 250 rpm shaking. From the overnight culture, 0.1% of the inoculum was inoculated in 10 ml of LB and grown until an OD_600_ of 0.5 to 0.6 was reached. At this point the culture was maintained at 15°C for 30 min and IPTG was added to a final concentration of 1 mM. The culture was grown overnight at 15°C. The cells were harvested by centrifugation at 10,000 rpm for 10 min and subjected to protein purification.

### Purification of recombinant protein ROP8

The histidine tagged recombinant protein was purified through native purification system (Invitrogen, USA) using nickel nitrilotriacetic acid (Ni-NTA) (Qiagen, USA). Briefly, the cell pellet was suspended in binding buffer and solubilized by sonication on ice using a high intensity pulse for 15 mins. The lysate was then separated by centrifugation at 10,000 rpm for 30 min and incubated with Ni-NTA resin for 3 h at room temperature. The resin was washed with binding buffer containing increasing concentrations of Immidazole (5, 10 and 15 mM) to remove all contaminating proteins. The recombinant protein was eluted at 200 mM immidazole concentration. The purity of the proteins was analysed by running a 12% SDS-PAGE. The concentration of the purified recombinant proteins was estimated by the Bradford method (Bio-Rad, USA) and stored at −80°C in aliquots till further use.

### Western blot analysis of ROP8 recombinant protein

Proteins were separated according to their molecular weight using a 12% SDS-PAGE gel. The proteins on the gel were transferred to a polyvinylidene difluoride (PVDF) membrane (Bio-Rad, USA). The membrane was blocked with blocking agent (5% non-fat milk powder in TBS) and washed three times with TBS and TBS-T for 5 min. This was followed by incubation with primary antibody (1:250) in 2.5% blocking agent in TBS for 2 h at room temperature and subjected to washing. The membrane was then incubated with anti-Human IgG (1:2500) for 1 h with shaking, washed and incubated with SAAP (1:2500) for 1 h. Finally, the membrane was washed and the protein was detected using nitro blue tetrazolium/bromochloroindolyl phosphate (NBT/BCIP) as the substrate.

### Analysis of ROP8 protein sensitivity and specificity

Purified rROP8 were evaluated in Western blot assays using serum samples from three groups of human toxoplasmosis cases. Group 1 consisted of patients with early acute toxoplasmosis (IgM positive, IgG negative; n = 25); Group 2, consisted of patients with acute toxoplasmosis (IgM positive, IgG positive; n = 30); Group 3, consisted of patients with chronic toxoplasmosis (IgM negative, IgG positive; n = 50) and Group 4, consisted of toxoplasmosis negative control healthy human serum samples (IgM negative, IgG negative; n = 100). These samples were obtained from the Diagnostic Laboratory at the Department of Parasitology, University of Malaya, and grouped based on Captia™ *Toxoplasma gondii* IgG and *Toxoplasma gondii* IgM kits (Trinity Biotech, Ireland). Specificity of the recombinant protein was also tested with sera of patients diagnosed as having different infections, namely, amoebiasis (2 samples), cysticercosis (4 samples), filariasis (3 samples), and toxocariasis (2 samples). After the western blot analysis, sensitivity and specificity of rROP8 protein was calculated and tabulated in Table 1. Sensitivity was calculated as (number of true positive results)/(number of true positive results + number of false-negative results), and specificity was calculated as (number of true negative results)/(number of true negative results + number of false-positive results).

**Table 1:** Summary of western blot results obtained from recombinant rhoptry protein 8 detected with human serum samples

**Sample group**	**Number of human serum samples**	**Western blot**			
		**Positive**		**Negative**	
		**No.**	**%**	**No.**	**%**
1 (early acute: IgG−, IgM+)	25	23	90	2	10
2 (acute: IgG+, IgM+)	30	23	92	3	8
3 (chronic: IgG+, IgM−)	50	41	82	9	18
4 (toxoplasmosis negative: IgG−, IgM−)	100	6	6	94	94
Other infections					
Amoebiasis	2	0	0	2	100
Cysticercosis	4	0	0	4	100
Filariasis	3	0	0	3	100
Toxocariasis	2	0	0	2	100

## Results

### Cloning of the *ROP8* gene into the pCOLD I DNA vector

The *ROP8* gene (1728 bp) was amplified from tachyzoite genomic DNA and successfully cloned into the pCOLD I DNA vector. Positive colonies were confirmed by polymerase chain reaction (PCR) and sequencing using gene-specific primers. Sequencing results revealed 100% similarity with the previously published *ROP8* gene sequence. Finally, the verified recombinant plasmid was transformed into the BL21 (DE3) expression host.

### Expression and purification of rROP8

**Figure 1:**
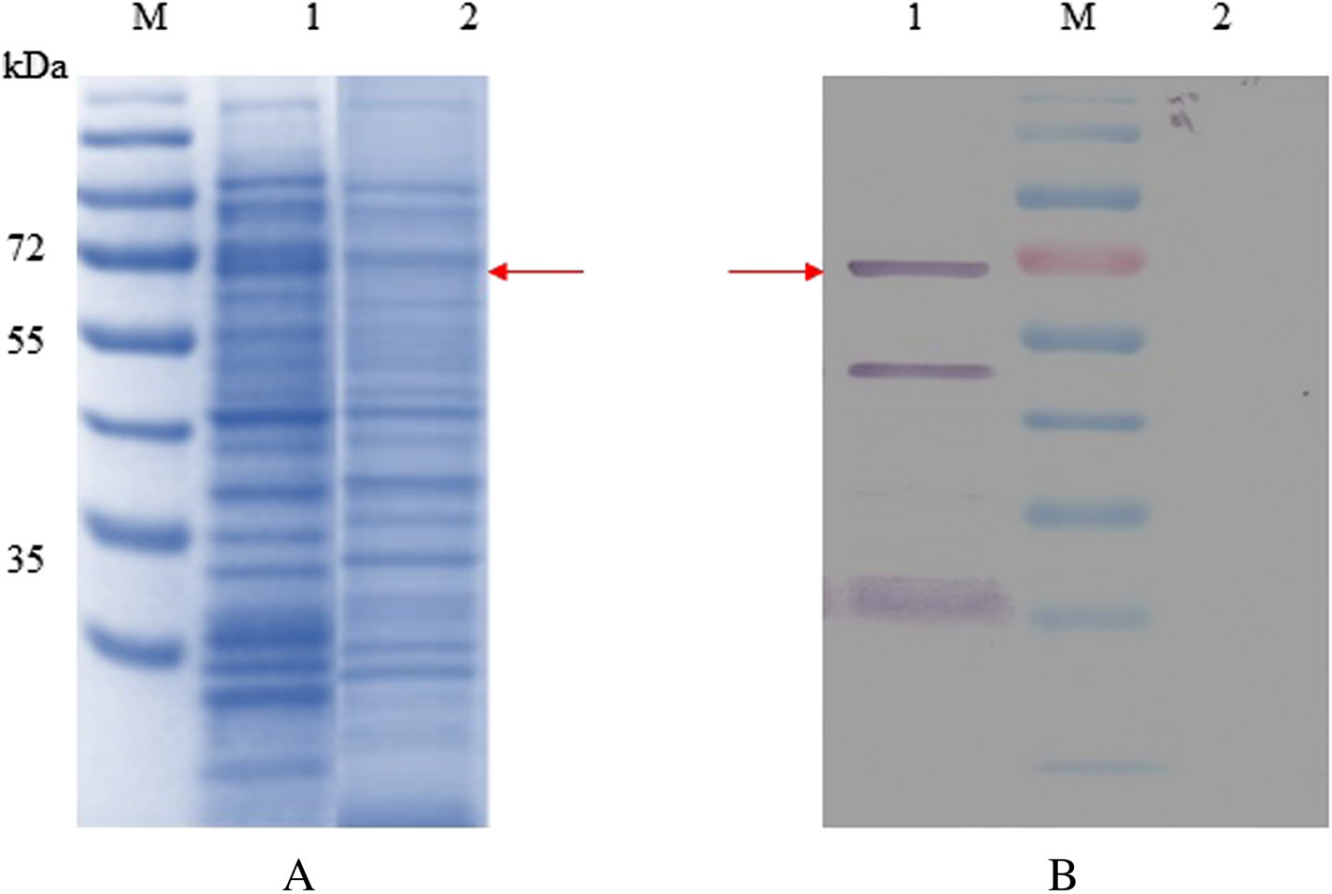
**SDS-PAGE analysis of expression of rROP8 in pCOLD I DNA vector. (A)** Coomassie brilliant blue stained, **(B)** Western blot probed with Toxoplasmosis -infected patient serum. Lane 1 is rROP8 protein, Lane 2 is vector control (pCOLD I DNA vector protein only). Arrow indicates the expression of rROP8 protein at approximately 67 kDa.

**Figure 2:**
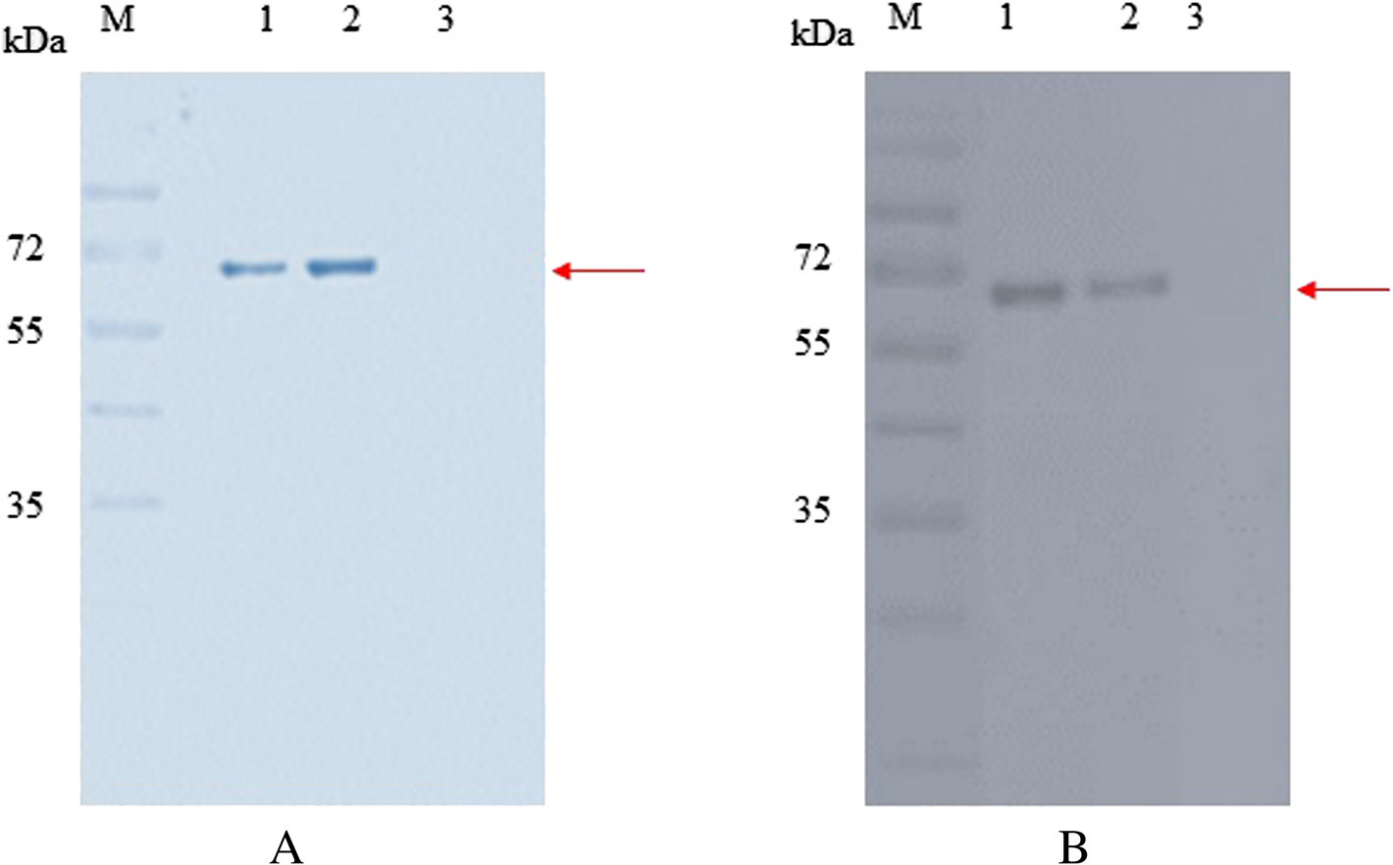
**SDS analysis of purified rROP8. (A)** Coomassie brilliant blue stained, **(B)** Western blot probed with *Toxoplasmosis* patient serum_._ Lane 1 and 2 are rROP8 protein. Lane 3 is vector control (pCOLD I DNA vector protein). Arrow indicates the purified rROP8 protein at approximately 67 kDa.

Expression of rROP8 protein was induced with 1 mM IPTG for 24 h. SDS-PAGE was subsequently performed to analyze the expressed rROP8, which appeared as an intense 67 kDa band that was absent from the negative control (vector only) (Figure 1A and 1B). The rROP8 protein was successfully purified under native conditions using Ni-NTA resin (Figure 2A). Purification of rROP8 was verified by western blot with both His tag-specific antibody (anti-His) and human serum from a *Toxoplasma*-infected patient (Figure 2B). In each case a 67 kDa band was observed, while nothing was detected in the vector control lane.

### Reactivity of human serum samples against rROP8 protein

**Figure 3:**
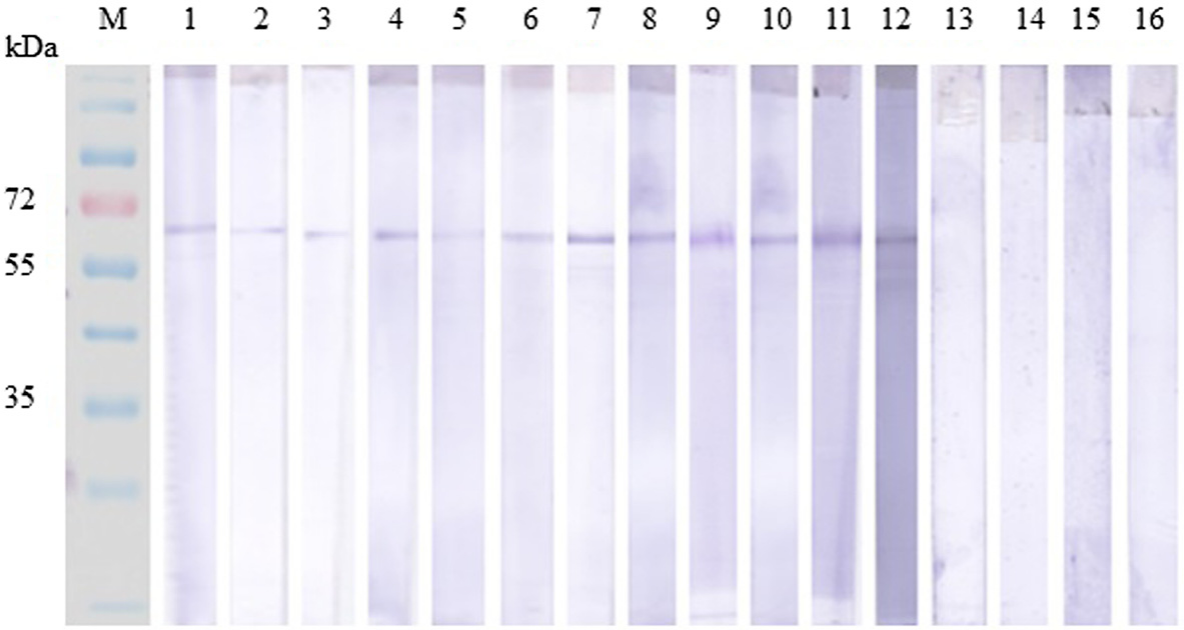
**Detection of rROP8 with patient sera infected by Toxoplasmosis.** Each Western Blot strip of the purified rROP8 was tested with serum from each category. Four samples from each category are shown here. Lanes 1 to 4 represents results of group 1 serum samples (early acute IgG−, IgM+), lane 5 to 8 represents results of group 2 serum samples (acute: IgG+, IgM+), lane 9 to 12 represents results of group 3 serum samples (chronic: IgG+, IgM−), lanes 13 to 16 represents results of group 4 serum samples(toxoplasmosis negative: IgG−, IgM−) as a control group.

*Toxoplasma*-infected human serum samples were used to evaluate the sensitivity and specificity of rROP8 as a diagnostic marker by western blot. For this analysis, recombinant protein was tested against three different infected serum groups: early acute (IgG^−^, IgM^+^), acute (IgM^+^, IgG^+^), and chronic (IgG^+^, IgM^−^). Non-infected human serum samples were employed as a negative control. In addition, rROP8 was tested against human sera corresponding to other non-*Toxo* parasitic infections, such as toxocariasis, filariasis, amoebiasis, and cysticercosis. We observed that rROP8 protein yielded the following sensitivities with regard to serological detection of *T. gondii*: 90% (23 of 25) for early acute, 92% (23 of 30) for acute, and 82% (41 of 50) for chronic infections (Table 1). Four positive results for each group are shown in (Figure 3). Considering the 100 toxoplasmosis-negative human serum samples, six samples were found to react with rROP8, yielding a specificity of 94% (6 of 100) (Table 1). None of the non-*Toxo* parasitic infection sera reacted with rROP8 protein.

## Discussion

In the present study, the *ROP8* gene was successfully cloned into a bacterial expression vector (pCOLD I DNA), and rROP8 protein was expressed in BL21 (DE3) host cells. One-step metal affinity chromatography was subsequently performed to purify rROP8, producing a low amount of pure recombinant protein per liter of bacterial culture. Notably, rROP8 production was found to be less efficient than other antigens that we have previously expressed and purified in our laboratory. Nevertheless, we retrieved sufficient quantities of rROP8 for testing its value as a diagnostic marker.

We tested the reactivity of three distinct groups of toxoplasmosis-infected human sera (i.e., early acute, acute, and chronic phase samples) against rROP8 by western blot (Table 1). Importantly, we observed no rROP8 cross reactivity when analyzing sera from other parasitic infections (i.e., amoebiasis, cysticercosis, filariasis and toxocariasis). We found that purified rROP8 antigen showed high sensitivity and specificity for the diagnosis of toxoplasmosis. In fact, our western blot analysis revealed a specificity of 96% and sensitivity for detecting IgG of 90%. This sensitivity was slightly higher when compared to ROP1 antigen, which was recently investigated with regard to the detection and differentiation of acute and chronic phase toxoplasmosis [22]. In addition, Van Gelder (along with other groups) evaluated recombinant ROP2 expressed in *E. coli*, as a marker for detection of *T. gondii*-specific antibodies in sera and found that recombinant ROP2 antigen detected IgG in 89 out of 98 toxoplasmosis-positive samples. A similar study was performed by Martin, who observed a sensitivity of 91% when using *E. coli*-expressed ROP2 to detect toxoplasmosis-specific IgG in human serum samples [23].

The major finding of this study is that rROP8 antigen represents a valid diagnostic marker for detecting toxoplasmosis via western blot assay. Notably, the western blot method is thought to be better than enzyme-linked immunosorbent assay (ELISA), especially considering that the likelihood of obtaining false positive results by western blot is much lower as compared to ELISA [24]. The superiority of the western blot assay over ELISA for screening human serum samples has also been confirmed in other studies. In fact, immunoblot was found to be more informative and less affected by sample degradation than ELISA, producing high confidence results that could be directly visualized [25]. Thus, given its high specificity and sensitivity, we have selected to use the western blot technique to evaluate the efficacy of rROP8 as a diagnostic marker for toxoplasmosis.

Our rROP8-related findings might be valuable for future development of diagnostic kits based on the immmunochromatographic test (ICT). The ICT represents the best serological assay for diagnosing infections, including toxoplasmosis compared to ELISA, although ELISA is more commonly used and considered to be simpler than other tests. Nevertheless, the ICT is more rapid, efficient, accurate, and low cost than ELISA [26]. In addition, ICT is advantageous because it can be used in the field (e.g., diagnosis of farm animals) [26].

One of the limitations of this study is failure to follow up with a repeat serology test on a second sample from the same patient to confirm the acute infection. By standard practice, early acute infection is confirmed by repeat sampling between 7–15 days and observing the IgG conversion [1]. Repeated sampling was not feasible in this study as patients were discharged immediately after treatment.

## Conclusion

Our findings demonstrate that rROP8 produced in *E. coli* can be used as an antigen for the detection of toxoplasmosis-specific antibodies (IgG and IgM) by western blot assay. Thus, rROP8 represents a valid diagnostic marker for differentiating between the distinct stages of toxoplasmosis infection. In fact, the rROP8 based detection method yielded a 90% sensitivity and 94% specificity. Thus, rROP8 may represent a useful candidate antigen for the development of a novel ICT assay to detect toxoplasmosis. Furthermore, the *ROP8* gene could be considered for future toxoplasmosis vaccine development.

## Competing interests

The authors declare that they have no competing interests.

## Authors’ contributions

PS, MYF, RK and YLL contributed to the conception and design of the study, analyzed the results and wrote the paper. RM collected the samples and confirmed the diagnosis. All authors read and approved the final manuscript.
